# Epithelial ciliated beating cells essential for ex vivo ALI culture growth

**DOI:** 10.1186/s12890-017-0423-5

**Published:** 2017-05-03

**Authors:** Delphine Gras, Aurélie Petit, Jérémy Charriot, Lucie Knabe, Khuder Alagha, Anne Sophie Gamez, Céline Garulli, Arnaud Bourdin, Pascal Chanez, Nicolas Molinari, Isabelle Vachier

**Affiliations:** 1 UMR INSERM U1067 CNRS 7333, Aix Marseille University, Marseille, France; 2 Department of Respiratory Diseases, CHRU Montpellier, Montpellier, France; 3 U1046 INSERM, UMR9214 CNRS, Montpellier University, Montpellier, France; 4 Department of Respiratory Medicine, Assistance Publique Hopitaux de Marseille, Aix Marseille University, Marseille, France; 5 Institut Montpelliérain Alexander Grothendieck, CNRS, UM, Montpellier, France; 6 Department of Statistics, CHRU Montpellier, Montpellier, France; 7 CHU Montpellier, Hôpital Arnaud de Villeneuve, 371 Av Doyen G Giraud, 34295 Montpellier Cx 5, France

## Abstract

**Background:**

Bronchial epithelium plays a key role in orchestrating innate and adaptive immunity. The fate of ex vivo airway epithelial cultures growing at the air liquid interface (ALI) derived from human endobronchial biopsies or brushings is not easy to predict. Calibrating and differentiating these cells is a long and expensive process requiring rigorous expertise. Pinpointing factors associated with ALI culture success would help researchers gain further insight into epithelial progenitor behavior.

**Methods:**

A successful ALI culture was defined as one in which a pseudostratified epithelium has formed after 28 days in the presence of all differentiated epithelial cell types. A 4-year prospective bi-center study was conducted with adult subjects enrolled in different approved research protocols.

**Results:**

463 consecutive endobronchial biopsies were obtained from normal healthy volunteers, healthy smokers, asthmatic patients and smokers with COPD. All demographic variables, the different fiber optic centers and culture operators, numbers of endo-bronchial biopsies and the presence of ciliated cells were carefully recorded. Univariate and multivariate models were developed. A stepwise procedure was used to select the final logistic regression model. ALI culture success was independently associated with the presence of living ciliated cells within the initial biopsy (OR = 2.18 [1.50–3.16], *p <* 0.001).

**Conclusion:**

This finding highlights the properties of the cells derived from the epithelium dedifferentiation process. The preferential selection of samples with ciliated beating cells would probably save time and money. It is still unknown whether successful ALI culture is related to indicators of general cell viability or a purported stem cell state specifically associated with ciliated beating cells.

**Electronic supplementary material:**

The online version of this article (doi:10.1186/s12890-017-0423-5) contains supplementary material, which is available to authorized users.

## Background

Bronchial epithelial cells represent the primary airway defense barrier against inhaled pathogens and particles [1, 2]. This epithelium plays a crucial role in maintaining airway homeostasis by regulating innate and acquired immunity [3] through the production of a wide range of cytokines, chemokines and mediators. The bronchial epithelium involves a pseudostratified organization of basal, ciliated, goblet and other less common types of cells [1, 4]. These different cell types appear to be essential for proper function [1, 2].

The majority of initial studies compared epithelial content in experiments performed on biopsy, lung resection or autopsy specimens, but only recently on bronchoscopy specimens [5]. Epithelial cell culture techniques have been well documented for many years, including studies on cell lines (immortalized or not) and on primary cells obtained from volunteers who submitted to brushings [6] or forceps biopsies [7] (used as primary cells or after monolayer cultures). Studies with cell lines are easy, reproducible and not very expensive, whereas those carried out with primary cultures are hampered by the high cost, between-donor variability, the dissociation process, as well as the limited number of cells suitable for experiments. In monolayer cultures, the number of passages is critical and cells clearly undergo a dedifferentiation process and lose their phenotype [8]. This has led to the development of new cell-line systems involving a monolayer culture followed by an air-liquid interface (ALI) using an appropriate medium to preserve cell phenotypes [9, 10]. This human bronchial epithelial cell (HBEC) model can generate polarized pseudostratified epithelium composed of most known cell types [11]. This culture system provides a useful tool for the in vitro study of airway epithelial biological features and cell differentiation [11, 12], including functional studies of specific differentiated cells (i.e. ciliary beating, mucus secretion and cytokine production).

Implementation of the ALI technique is currently the focus of an increasing number of publications because it reproduces a well differentiated airway epithelium with distinct, functional cells [13]. Many factors may contribute to the success or failure of ALI cultures, including initial sample types and amounts, underlying disease, sample handling, techniques and media [14, 15].

In this study, we aimed to identify potential predictive factors for obtaining a successful ALI culture that could be used in future experiments. We investigated these variables in all experiments performed in our laboratory on bronchial biopsies obtained from healthy volunteers, smokers, asthmatic (mild, moderate, and severe) subjects and COPD patients.

## Methods

### Subjects

Four hundred sixty-three adults were consecutively included from March 2009 to July 2013 at the *Hôpital Arnaud de Villeneuve* (Montpellier, France) and the *Assistance Publique des Hôpitaux de Marseille* (Marseille, France). They all signed an informed consent form in order to participate in a study approved by the ethics committees of our institutions for the purpose of a specific biomedical research project on epithelial pathophysiologic mechanisms.

Controls (*n =* 83) and smokers (*n =* 65) without COPD (no asthma, no allergies, normal chest X-ray and pulmonary function tests) underwent bronchoscopy for various purposes (foreign body removal, suspected but unconfirmed hemoptysis or peripheral nodules (<10 mm) found during CT scan examination) and had normal macroscopic airway appearance. Smokers were defined by a smoking status greater than 10 pack-years.

Asthma diagnosis (*n =* 175) was based on the assessment of clinical features consistent with asthma and evidence of variable expiratory airflow obstruction during functional testing (FEV_1_ increase of at least 12% and 200 ml after inhalation of 200 μg of salbutamol or after 4 weeks of oral corticosteroid treatment, an average daily diurnal PEF variability < 10%). Asthma severity was evaluated according to the current global initiative for asthma guidelines (http://ginasthma.org/). Patients with severe asthma (*n =* 124) also met the American Thoracic Society criteria for refractory asthma [16]. Asthmatic patients had been free of respiratory infections and asthma exacerbations for at least 6 weeks at the time of inclusion. They were current non-smokers or had a smoking history of less than 5 pack-years [17].

COPD diagnosis (*n =* 130) was based on evidence of non-reversible obstructive airflow FEV_1_/FVC < 0.7, FEV_1_ improvement after inhaling 200 to 400 μg of albuterol below 12% and 200 ml, and a smoking history greater than 10 pack-years without any evidence of any alternative diagnosis according to the best standards of care (i.e. no history of any other respiratory disease based on clinical examination or computed tomography scan findings) [18].

### Endobronchial biopsy

Flexible bronchoscopies were usually performed under local anesthesia and two biopsies from each donor were removed using alligator forceps (Olympus) on a subsegmental bronchus of the left lower lobe, as previously described [19]. Briefly, following premedication with subcutaneous atropine (0.25 mg) and midazolam (5 mg), local naso-pharyngeal anesthesia was implemented using lidocaine (1 – 5%, with a maximum individual dose of 300 mg). Then a fiber optic bronchoscope (Olympus BF20) was nasally inserted into the trachea.

### Cell culture

Primary human bronchial epithelial cells were obtained from bronchial biopsy specimens and cultured under ALI conditions, as previously described [14]. Briefly, bronchial epithelial biopsy tissue was dissociated and suspended in bronchial epithelial growth medium (Lonza). After an expansion phase in monolayers, cells were plated on uncoated nucleopore membranes (24-mm dia., 0.4-mm pore size, Transwell Clear) in a 1:1 mixture of bronchial epithelial growth medium and Dulbecco’s modified Eagle’s medium (Lonza) applied only at the basal side to establish the ALI. Cells were cultured for 28 days to obtain a polarized and differentiated cell population with a mucociliary phenotype.

### Immunofluorescence

ALI cultured cells were fixed in situ on inserts and transferred to glass slides for analysis. Cells were fixed using 10% formalin and blocked/permeabilized with PBS, 10% donkey serum, 1% BSA, and 0.1% Triton-X. Cells were incubated with appropriate primary antibodies at 4°C overnight (mouse monoclonal anti-β-tubulin IV (ONS.1A6, Sigma-Aldrich), mouse monoclonal anti-Muc5AC (45M1, Abcam) and goat polyclonal anti-p63 (S16, Santa Cruz Biotechnology)). Alexa Fluor® 488, Alexa Fluor® 555 or Alexa Fluor® 647 secondary labelling (Invitrogen) was applied for 1 h at room temperature and incubated with DAPI before mounting. Negative controls were incubated with secondary labelling antibodies only. Cells were visualized under a Zeiss axioimager microscope using axiovision software (Zeiss).

### Data format and statistical analysis

We defined three successful culture steps. First, we considered that a monolayer culture was successful when cells were growing in monolayers but did not reach the ALI transfer stage (denoted “ML”). Then we considered that the ALI passage was successful when cells were able to reach the ALI step without a successful 28-day culture (denoted“pALI”). Finally, ALI culture was considered successful when a pseudostratified epithelium was obtained with the presence of at least basal, ciliated and goblet cells and without any contamination after 28 days of ALI culture (denoted “ALI 28d”).

Continuous parametric data are presented as means ± standard deviation (SD), and categorical variables as numbers and percentages. We used the *χ* ^2^ test for categorical variables. A multivariate analysis was conducted using a Cox regression model to determine independent factors of success, in which we included all variables associated with a *p-*value of below 0.20 in the univariate analysis. Then a stepwise procedure allowed us to obtain the final multivariate model. Survival curves were estimated with the Kaplan Meier method and the log-rank test was used. Statistical tests were performed using R (version 3.1.0 (2014-04-10) R Foundation for Statistical Computing) software. The significance level was set at *p* ≤ 0.05.

## Results

### Patient characteristics

Table 1 summarizes the demographic and clinical characteristics of subjects included in the study.

**Table 1:** Demographic and clinical characteristics of subjects

	Overall *N =* 463	Control *N =* 83	Smokers *N =* 65	COPD *N =* 130	Mild-Mod A *N =* 61	Severe A *N =* 124
Sex F (n, %)	214 (47)	49 (60)	20 (31)	22 (17)	39 (66)	84 (68)
Age (mean ± sd)	54.7 ± 14.4	50.1 ± 17.0	53.5 ± 14.2	62.4 ± 10.2	52.5 ± 14.6	51.5 ± 13.1
BMI	24.3 ± 4.7	23.5 ± 4.6	24.9 ± 4.8	23.8 ± 4.2	22.4 ± 2.8	27.9 ± 6.3
FEV_1_ %	78.8 ± 22.7	97.8 ± 12.9	96.4 ± 18.4	64.2 ± 20.5	89.6 ± 15.0	71.3 ± 19.1
FVC %	94.8 ± 21.7	98.2 ± 19.4	103.5 ± 21.9	88.7 ± 20.8	112.4 ± 15.4	83.5 ± 14.5
FEV1/FVC	64.8 ± 14.0	78.5 ± 9.5	75.6 ± 6.0	55.7 ± 11.1	69.8 ± 10.7	59.1 ± 13.7
ICS (n, %)	172 (37)	0 (0)	0 (0)	33 (25)	38 (62)	101 (81)
OCS (n, %)	70 (15)	6 (7)	4 (6)	4 (3)	2 (3)	54 (44)
SABA (n, %)	102 (22)	0 (0)	0 (0)	15 (12)	15 (25)	71 (57)
LABA (n, %)	165 (36)	0 (0)	0 (0)	36 (28)	28 (46)	99 (80)
LAMA (n, %)	23 (5)	0 (0)	0 (0)	18 (24)	1 (2)	3 (2)
MTP/MRS	204/259	34/49	64/1	82/48	9/52	15/109
Biopsy Number (mean ± sd)	1.57 ± 1.09	1.58 ± 1.16	2.88 ± 0.96	1.90 ± 1.28	1.25 ± 0.85	1.07 ± 0.40
Biopsies grown by a senior operator (*n*, %)	259 (70)	48 (77)	32 (49)	69 (53)	37 (79)	73 (85)

As expected, FEV_1_ was significantly decreased in severe asthma and COPD patients, while FEV_1_/FVC was decreased in COPD. Moreover, treatment intake is more important in severe asthma and COPD than in mild-moderate asthmatics and controls or smokers.

Biopsy cultures from asthmatic patients were mainly performed and obtained at the Marseille (MRS) center, while those for COPD and smokers were mainly from Montpellier (MTP).

### HBEC cultures

Overall, 65% of biopsy cultures were successfully grown as monolayers in flasks and no differences in success were noted between groups of subjects. The major reason for unsuccessful monolayer culture was bacterial and/or fungal contamination at an early stage. In such cases, the cultures were excluded in order to avoid any further contamination within the incubator. The other finding at this stage was the incapacity of cells to adhere and grow. In such cases, cells were left in culture for up to 2 weeks and were then stopped.

**Fig. 1:**
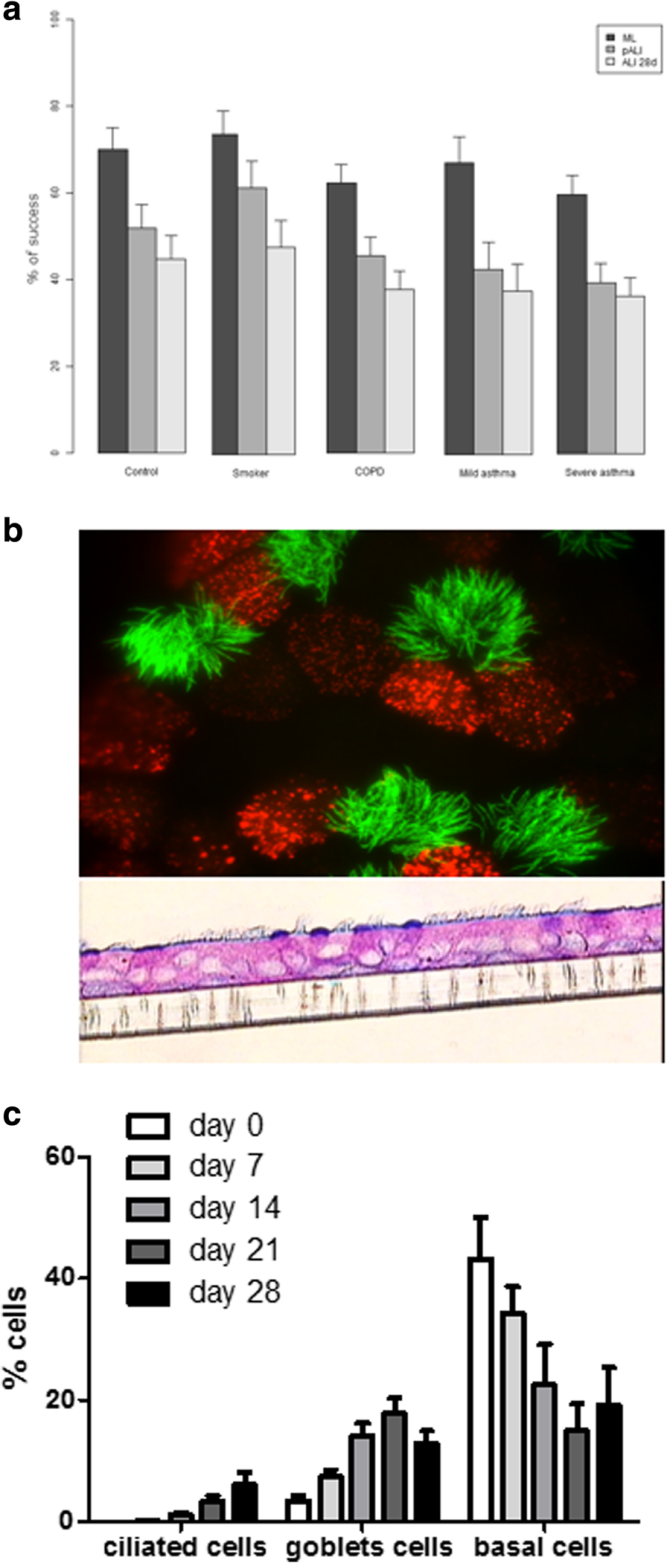
**a** Percentage of success in obtaining a monolayer culture (ML), the filter passage (pALI) and complete success at 28 days of ALI culture (ALI 28d) in controls, smokers, COPD, mild and severe asthma samples. **b** A representative figure of an ALI culture at 28 days differentiation. **c** Percentage of epithelial cells (ciliated, goblet and basal cells) during the differentiation process at 28 days ALI culture using immunofluorescence with specific antibody against β-tubulin IV, Muc5AC and p63, respectively

Thereafter, 47% of samples were transferred into transwells for the first liquid-liquid interface step. Finally 40% of the total initial biopsy specimens were successful in ALI culture at 28 days and could be used for further experiments (Fig. 1a). At this stage, a well differentiated pseudostratified epithelium was obtained with the presence of at least basal, ciliated and goblet cells (Fig. 1b). The percentage of ciliated and goblet cells increased from day 0 to day 28, whereas the percentage of basal cells did not change or tended to decrease (Fig. 1c).

### Factors associated with ALI culture at 28 days

Univariate analysis indicated that 28-day ALI culture success was associated with the absence of inhaled corticosteroid use (HR = 0.64 [0.45–0.92]; *p =* 0.02), long acting β agonist use (HR = 0.62 [0.43–0.88]; *p =* 0.01), and the presence of beating ciliated cells within the initial bronchial biopsy (HR = 2.18 [1.50–3.16]; *p <* 0.001). Univariate analysis results are tabulated in the supplemental data file (see Additional file 1).

According to multivariate analysis, the success in obtaining a useful 28-day ALI culture was associated only with the presence of beating ciliated cells (supplemental online cell videos obtained from biopsy samples are provided; see Additional files 2 and 3).


Additional file 2: Video S1. Showing cells obtained from the biopsy sample with the presence of beating ciliated cells. (AVI 1483 kb)



Additional file 3: Video S2. showing cells obtained from the biopsy sample with the presence of beating ciliated cells. (AVI 785 kb)


### Success with and without the presence of beating ciliated cells in the initial bronchial biopsy

**Fig. 2:**
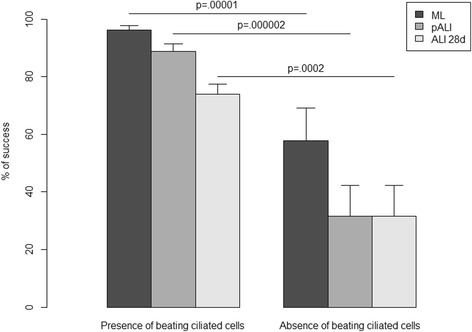
Percentage of success in obtaining a monolayer culture (ML), the filter passage (pALI) and complete success at 28 days of ALI culture (ALI 28d) in bronchial biopsies from all patients significantly differed between cultures obtained from initial biopsies where beating ciliated cells were present versus not (*p =* .00001, .000002 and .0002, respectively, according to a *χ* ^*2*^ *test*)

We obtained a significantly higher percentage of success in generating a well pseudostratified epithelium in 28-day ALI cultures for all pathology conditions (*p =* 0.00001, 0.000002 and 0.0002 according to the *χ* ^2^ test for ML, pALI and ALI 28d, respectively), when beating ciliated cells were present at the beginning of the process, just after the bronchoscopy procedure (Fig. 2).

**Fig. 3:**
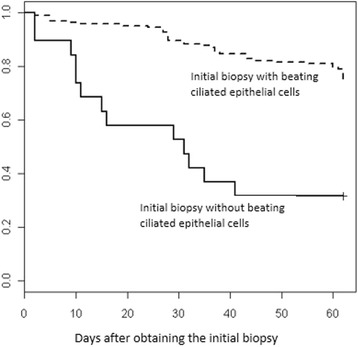
Cox model applied to assess the time of success for cultures differentiated with the presence or not of beating ciliated epithelial cells

In order to test the effects of the presence of beating ciliated cells in the initial biopsy, we applied a Cox proportional hazards model (Fig. 3). Clearly, the presence of beating ciliated cells in the initial biopsy is associated with the success rate.

## Discussion

In the present study, we aimed to describe our expertise in the development of human bronchial epithelial cell ALI cultures obtained from human bronchial biopsies, and to identify potential predictive factors associated with the success of this process after 28 days of culture. We consecutively included the 463 bronchial biopsies taken in order to develop ALI cultures at out two specialized centers in control subjects as well as in pathological conditions. We drew up an exhaustive list of patient characteristics and procedure details which could interfere with the success rate. The main finding of the multivariate analysis was that the presence of ciliated beating epithelial cells at the beginning of the procedure, just after the bronchial biopsy specimen was obtained, was the only parameter associated with the percentage of success in obtaining a well pseudostratified epithelium in a 28-day ALI culture.

Bronchial epithelium is characterized by the presence of differentiated epithelial cell types organized as a pseudostratified epithelium. Currently, and since the beginning of in vitro studies on human bronchial epithelial cells, cultures come mainly from the sources described hereafter [8]. Carcinoma-derived cells formed confluent polarized monolayers but lacked pseudostratification and the presence of ciliated cells, even though this effect might be counteracted when cultured in ALI [20]. Virus transformed cell lines form polarized multilayer cultures but often lack mucus production and relevant genes might be altered or not expressed [21]. Finally, primary cells were found to result in well differentiated cultures representative of a human bronchial phenotype but with a limited lifespan. Culturing at an ALI is essential for polarity and differentiation in these cells [11].

For 10 years now, we have thus focused on finding technologies that result in well pseudostratified structures as well as other in vivo-like differentiation characteristics. Primary bronchial epithelial cells specifically showed a mucociliary differentiation pattern comparable to that noted in vivo [22]. This technique for obtaining in vitro epithelium is now widely used, but the model has yet to be clearly characterized, even though the persistence of the pathological phenotype has been well described [14, 15]. The generation of differentiated cultures of normal human bronchial epithelial cells is a time- and labour-intensive procedure, especially when producing a large number of cell cultures for analysis [23]. This is why we wanted to describe optimal conditions for reliably obtaining epithelial cultures for further experiments, and to address a list of factors which could predict this success. Our study does not address expected growth patterns for bronchial brushing samples, which is another source of cells already in use [6]. This study was performed on all consecutive samples obtained in our two specialized centres from 2009 to 2013. The results revealed that only 40% of the samples were successfully cultured, which is a low rate. However, the latter reflects the real world difficulties associated with training and practice. In follows that all predictive conditions which might further increase yield are being sought.

In order to better describe the dynamic changes that occur during the culture of differentiated epithelia, we were able—in a subset of culture wells from different subject phenotypes—to count the number of ciliated, goblet and basal cells by using specific labelled antibodies. In the initial mucociliary differentiation step (day 0 of ALI culture), we detected a population of basal epithelial stem cells (p63^+^) and undifferentiated cells, which then decreased over time. It has been reported that basal stem/progenitor cells are involved in the regeneration of human bronchial epithelium [24]. Basal and undifferentiated epithelial cells decreased during mucociliary differentiation, whereas goblet and ciliated cells were found to increase from day 7 to day 14, respectively, to reach maximal differentiation at 28 days. These findings are in accordance with previous studies describing the mucociliary differentiation of normal human tracheobronchial cells [25].

Our major finding was the partial explanation for 28-day culture success as being due to the presence, in the original biopsy, of ciliated beating cells. One of the limitations of our study was that we did not assess the histological features of the initial biopsy. We chose instead to use all of the initial material obtained from the bronchoscopy in order to increase culture success. It is still unclear whether this result reflects the true presence of ciliated cells, which may act as progenitors, or the fact that visual beating is an index of better viability of the original cells. Under physiological conditions, normal adult human bronchial epithelium turnover is relatively slow, i.e. approximately every 1 to 4 months [26], and the basal cells are relatively quiescent. in vivo lineage-tracing studies in pseudostratified mucociliary epithelium from mouse trachea have shown that basal cells can function as classical stem cells and both self-renew and give rise to ciliated and secretory cells [27]. Notch signalling promotes this differentiation, favouring the production of ciliated cells and high levels of secretory cell fate [28]. Therefore, differentiated cells are commonly thought to send back signals to their respective stem and progenitor cells to regulate their proliferation and differentiation [29]. The term dedifferentiation was used to suggest that differentiated epithelial cells revert to a previous developmental stage before their subsequent differentiation into an alternative cell fate [30]. These authors highlighted the existence of multiple cellular reservoirs of regenerative capacity, which may enable a more effective reparative response.

Moreover, light microscopy observations readily revealed the presence of beating cilia when the biopsy was viewed just before cell detachment at the outset of monolayer culturing. The verification of cell viability could easily relate to better cell behaviour leading to increased cell adherence for further dedifferentiation/differentiation processes.

The biology and relevance of bronchial epithelium stem cells remain unclear. Identification of the cellular and molecular mechanisms of bronchial stem cells involved in repair, proliferation, and mucociliary differentiation under normal and pathological conditions will provide further insight into cellular differentiation with regard to the development of new therapeutic strategies or airway reconstruction methods for obtaining, for instance, artificial trachea.

## Conclusion

This is the first study to characterize the properties of cells derived from the bronchial epithelium dedifferentiation process. The presence of ciliated beating epithelial cells was the only parameter associated with success in obtaining a well pseudostratified epithelium in a 28-day ALI culture. The latter finding will influence our practice towards the preferential selection of samples when ciliated beating cells in order to save time and money.

## Additional files

Additional file 1: Table with the univariate analysis results. (DOCX 14 kb)

## Abbreviations

ALI 28d: Air liquid interface at 28 days
ALI: Air liquid interface
BMI: Body mass index
COPD: Chronic obstructive pulmonary Disease
CT scan: Computer tomography
FEV1: Forced expiratory volume in 1 second
FVC: Forced vital capacity
HBEC: Human bronchial epithelial cell
HR: Hazard ratio
ML: Monolayer
MRS: Marseille
MTP: Montpellier
pALI: Air liquid interface passage
PEF: Pulmonary expiratory function
