# Supplementary material for: Epithelial ciliated beating cells essential for ex vivo ALI culture growth
Source: BMC Pulm Med. 2017 May 3;17:80. doi: 10.1186/s12890-017-0423-5 (PMC5415749; doi:10.1186/s12890-017-0423-5)
Supplement: Supplementary file 3 — Table with the univariate analysis results. (DOCX 14 kb) [file 12890_2017_423_MOESM1_ESM.docx]

Additional File 1 Table: Univariate analyses

|  | Overall | Control | Smokers | COPD | Mild-Mod A | Severe A |
| --- | --- | --- | --- | --- | --- | --- |
| Age | 1.00 [0.99-1.01, 0.79 | 0.99 [0.97-1.01] 0.30 | 1.00 [0.97-1.02] 0.70 | 0.99 [0.97-1.02] 0.61 | 1.00 [0.97-1.02] 0.69 | **1.02 [1.00-1.04]**  **0.02** |
| Sex | 0.87 [0.68-1.10] 0.24 | 0.83 [0.45-1.51], 0.55 | 0.59 [0.26-1.31], 0.18 | 0.71 [0.39-1.32, 0.27 | 0.66 [0.34-1.27] 0.22 | 1.20 [0.74-1.93] 0.45 |
| Tobacco status | 1.00 [0.68-1.46] 0.99 | 0.88 [0.33-2.39] 0.81 | NA | 0.86 [0.12-6.19] 0.88 | 1.55 [0.52-4.64] 0.43 | 1.41 [0.54-3.65] 0.48 |
| Body Mass Index | 0.96 [0.91-1.02] 0.21 | 1.12 [0.95-1.32] 0.14 | **0.87 [0.79-0.96]**  **0.004** | **1.18 [1.01-1.37]**  **0.03** | 1.06 [0.79-1.41] 0.71 | 0.95 [0.84-1.08] 0.45 |
| FEV1/FVC | 1.01 [1.00-1.03] 0.17 | 1.06 [0.98-1.15] 0.12 | 1.02 [0.95-1.11] 0.55 | 1.02 [0.99-1.04] 0.31 | 0.99 [0.92-1.07] 0.82 | 0.98 [0.92-1.05] 0.60 |
| FEV1 | 1.01 [1.00-1.01] 0.07 | 1.01 [0.98-1.04] 0.56 | 1.01 [0.98-1.03] 0.64 | 1.01 [1.00-1.02] 0.21 | **1.04 [1.01-1.07]**  **0.01** | 1.00 [0.98-1.01] 0.66 |
| FVC | 1.01 [ 0.99-1.02] 0.22 | 1.01 [0.97-1.04] 0.68 | 1.00 [0.98-1.03] 0.73 | 1.01 [0.99-1.03] 0.23 | 0.97 [0.91-1.04] 0.43 | 1.02 [0.97-1.07] 0.51 |
| Inhaled steroids | **0.64 [0.45–0.92]**  **0.02** | NA | NA | 0.58 [0.33-1.02] 0.06 | 1.03 [0.24-4.38] 0.96 | 0.001 [0-999]  1 |
| Oral steroids | 0.82 [0.57-1.20], 0.32 | **0.16 [0.03-0.88]**  **0.03** | 0.83 [0.25-2.73] 0.77 | 1.23 [0.30-5.12] 0.77 | 1.99 [0.23-17.33]  0.50 | 1.04 [0.36-2.93] 0.95 |
| Short acting β-agonist | 0.87 [0.63-1.20] 0.39 | NA | NA | 0.72 [0.36-1.45] 0.38 | 1.29 [0.42-3.94] 0.66 | N0.99 [0.39-2.51]  0.99 |
| Long acting β-agonist | 0.62 [0.43-0.88]  0.01 | NA | NA | 0.63 [0.37-1.10] 0.11 | 0.86 [0.20-3.71] 0.84 | 0.001 [0-999]  1 |
| Long acting muscarinic | 0.67 [0.39-1.15] 0.17 | NA | NA | 0.67 [0.34-1.31] 0.25 | 0.25 [0.03-2.51]  0.30 | 0.85 [0.17-4.12] 0.84 |
| Center (Marseille) | **0.57 [0.42-0.76]**  **<0.001** | 1.74 [0.94-3.22] 0.07 | 0.22 [0.03-1.65], 0.23 | **0.50 [0.28-0.91]**  **0.02** | 0.90 [0.38-2.15] 0.81 | 0.96 [0.48-1.92] 0.91 |
| Culture operator | 1.24 [0.93-1.64] 0.14 | 1.15 [0.52-2.55] 0.73 | 1.93 [0.96-3.88]  0.06 | **2.05 [1.09-3.83]**  **0.03** | 0.72 [0.28-1.90] 0.50 | 0.83 [0.38-1.84] 0.64 |
| Biopsy number | 1.09 [0.96-1.25] 0.17 | 1.20 [0.88-1.63] 0.21 | 0.99 [0.59-1.66] 0.98 | 1.09 [0.87-1.37] 0.44 | 0.92 [0.65-1.29] 0.65 | 0.76 [0.45-1.34] 0.41 |
| Presence of ciliated beating cells | **2.18 [1.50-3.16]**  **<0.001** | 1.92 [0.67-5.56] 0.26 | **6.30 [4.62-8.58]**  **0.005** | **2.67 [1.31-5.43]**  **0.007** | 1.93 [0.45-8.23] 0.42 | 1.52 [0.85-2.71] 0.17 |

Results are presented as Hazard Ration [95% Confidential Intervals] and p-value.

NA : Non Applicable
